# Losses Motivate Cognitive Effort More Than Gains in Effort-Based Decision Making and Performance

**DOI:** 10.3389/fnhum.2020.00287

**Published:** 2020-07-17

**Authors:** Stijn A. A. Massar, Zhenghao Pu, Christina Chen, Michael W. L. Chee

**Affiliations:** Sleep and Cognition Laboratory, Centre for Sleep and Cognition, Yong Loo Lin School of Medicine, National University of Singapore, Singapore, Singapore

**Keywords:** cognitive effort, effort discounting, loss aversion, framing effect, motivation, sustained attention, N-Back, pupillometry

## Abstract

Human behavior is more strongly driven by the motivation to avoid losses than to pursue gains (loss aversion). However, there is little research on how losses influence the motivation to exert effort. We compared the effects of loss and gain incentives on cognitive task performance and effort-based decision making. In three experiments, participants performed a cognitively effortful task under gain and loss conditions and made choices about effort expenditure in a decision-making task. Results consistently showed significant loss aversion in effort-based decision making. Participants were willing to invest more effort in the loss compared to the gain condition (i.e., perform a longer duration task: Experiments 1 and 2; or higher task load: Experiment 3). On the other hand, losses did not lead to improved performance (sustained attention), or higher physiological effort (pupil diameter) in Experiments 1 and 2. In Experiment 3, losses did enhance working memory performance, but only at the highest load level. Taken together, these results suggest that loss aversion motivates higher effort investment in effort-based decision-making, while the effect of loss aversion during a performance may depend on the task type or effort level.

## Introduction

Motivation can be seen as the willingness to exert effort in the pursuit of a goal or outcome (Chong et al., 2016; Pessiglione et al., 2017). Exerting effort can aid performance by mobilizing cognitive or motor resources, leading to faster and/or more accurate responses (Manohar et al., 2015). It is thought, however, that such resource mobilization is costly (Kool et al., 2010), and optimal behavior relies on a constant weighing of effort-costs against the expected value of the outcomes (Kurzban et al., 2013; Westbrook and Braver, 2015). The higher the value of the outcomes, the more likely an individual is to expand the required effort.

How outcomes are valued depends on the way they are framed. A long literature on decision-making shows that people weigh avoiding losses more strongly than acquiring equivalent gains (loss aversion: Tversky and Kahneman, 1979). Accordingly, people are more willing to take risks (De Martino et al., 2006; Tom et al., 2007), or wait for an outcome (Xu et al., 2009; Blackburn and El-Deredy, 2013), if the outcome is framed as a loss rather than as a gain.

Although loss aversion is highly pervasive in decision making, most research has focused on decision making under risk, or on intertemporal choice. Very little research has been done on how losses affect the willingness to exert effort. The few studies exploring loss aversion in effort-based decision making have yielded inconclusive findings (Nishiyama, 2016; Lockwood et al., 2017; Byrne and Ghaiumy Anaraky, 2019; O’Brien and Ahmed, 2019; Chen et al., 2020). Similarly, studies examining cognitive performance under gain and loss incentives have not consistently found evidence for loss aversion (i.e., better performance and/or higher effort in loss incentive conditions compared to gains; Yechiam and Hochman, 2013; Belayachi et al., 2015; Paschke et al., 2015; Carsten et al., 2019).

In this study, we examined the effects of loss aversion on cognitive effort allocation. We tested this both in the context of performance (and associated physiology), and effort-based decision-making. Moreover, we tested this across different cognitive domains (sustained attention: Experiments 1 and 2; working memory: Experiment 3). In short, we found robust evidence for loss aversion in effort-based decision-making across all experiments. In contrast, loss aversion in performance was dependent on the cognitive domain and effort level.

## Experiment 1

### Methods

#### Participants

Thirty healthy participants were recruited from the student population [mean age (stdev) = 23.13 (3.07), 14 females]. Participants signed informed consent upon arrival in the lab. All procedures were approved by the Institutional Review Board (IRB) of the National University of Singapore.

#### Motivated Vigilance Task

**Figure 1:**
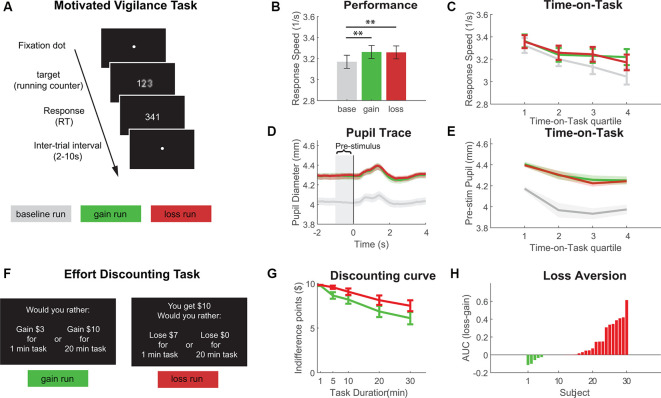
Results from Experiment 1 with **(A)** schematic of the Motivated Vigilance Task, **(B)** response speed in the vigilance task for baseline, gain and loss conditions, **(C)** Time-on-Task decline of response speed (Time-on-Task quartiles for illustrative purposes, statistical analysis was based on linear slopes), **(D)** target-locked pupil trace in the vigilance task (time = 0 indicates target presentation), **(E)** Time-on-Task decline of pre-stimulus pupil diameter (Time-on-Task quartiles for illustrative purposes only, statistical analysis was based on linear slopes), **(F)** example trials of the effort-discounting task, **(G)** effort-discounting curve, and **(H)** loss-gain difference in the area under the discounting curve (AUC; positive difference scores denote less discounting for loss vs. gain choices). **p < 0.01.

To assess sustained attention performance under gain and loss incentives, participants performed a Motivated Vigilance Task (see Figure 1A; Massar et al., 2016, 2019). Participants had to respond as quickly as possible a target (a running millisecond counter) by pressing a button. Target stimuli appeared at random intervals and were separated by a fixation dot. Upon response, the millisecond counter came to a stop, displaying the RT as performance feedback for 1 s. Each task run was 10 min, comprising approximately 80 targets.

Participants first performed an unincentivized baseline run, after which they performed two incentivized runs (gain and loss, order counterbalanced). In the gain run, they could earn 10¢ for each response that was faster than an individual reaction time (RT) criterion (their individual median RT in baseline; for full instruction see Supplementary Materials). Total earnings in this run could be up to approximately $8. In the loss condition, participants first received $8. They were then instructed instructed that they would lose 10¢ for every trial in which they responded slower than the criterion. The main performance outcome was response speed (1/RT). Furthermore, to obtain a physiological measure of cognitive effort, pupil diameter was during task performance using a Tobii X60 eye-tracker (Tobii AB, Danderyd, Sweden). Pupil diameter is reliably found to index effort during the cognitive performance (Kahneman, 1973), as it increases with task difficulty, motivation, and effort sensation). If losses would provoke higher effort exertion than gains, we would expect to see larger pupil diameter during the performance of the loss run. Following our earlier work (Massar et al., 2016), we extracted the average pupil size in a 1-second window before the target presentation as an index of sustained (tonic) effort (see Supplementary Materials for analysis details).

#### Effort Discounting Task

To examine the influence of gain vs. loss framing on effort-based decision making, participants performed an effort discounting task (Libedinsky et al., 2013). Participants were presented with a series of choice trials (see Figure 1F) in which they were given the option to earn a reward in return for further performance of the vigilance task for a specified duration. Each trial presented two choice options. One option offered a small reward in return for the performance of a short duration task (1 min). The other option offered a larger reward for a longer task duration (5, 10, 20, or 30 min). As sustained attention is perceived as more effortful with longer task duration, this can be thought of as a parametric increase in effort level. The discounting task was completed in two framing conditions, gain and loss (order counterbalanced).

In the gain condition, the larger reward for the longer duration task was fixed ($10). The smaller reward, for the short duration task, was dynamically updated after each trial, to approach the individual’s indifference point (i.e., the smaller reward at the lower effort that they found equally valuable as $10 for the higher effort level). Participants performed two runs of five trials per effort level and resulting indifference points were averaged per level.

In the loss condition, participants were first instructed that they could receive $10. They then completed the discounting task, in which they chose a shorter duration vigilance task and losing an amount of money, or longer duration task, and losing nothing. The amount to be lost was updated similarly as in the gain condition Initial amounts in the loss condition were pegged to those in the gain condition, such that the potential outcomes (the eventual reward, or what is left after subtracting the loss from the initial endowment) would be identical between both conditions. Two runs of five iterations per effort level were completed to obtain the average indifference points. After completion of the gain and loss runs, one trial was randomly drawn for execution (participants performed the vigilance task for the chosen duration and received the associated reward). To ensure participants did not make decisions based on their perceived (in)ability to perform, they were instructed that RT did not matter for this last run, but they should sustain effort throughout. To ensure decisions were not influenced by the temporal delay to reward, all participants were to stay in the lab for 30 min before receiving their rewards. During this time, they performed the vigilance task for the indicated duration and rested for the remaining time (see Supplementary Materials for full instructions).

### Results

#### Motivated Vigilance Task

There was a significant difference in response speed between the incentive conditions (see Figure 1B; *F*_(2,58)_ = 10.51, *p* < 0.001). Response speed was faster in both gain and loss conditions compared to baseline (gain: *t*_(29)_ = −3.68, *p* < 0.001; loss: *t*_(29)_ = −3.80, *p* < 0.001), but was equivalent between the gain and loss conditions (*t*_(29)_ = 0.197, *p* = 0.845). On average, 59.88% (± 12.55%) of responses were faster than RT criterion in the gain condition vs. 60.05% (± 13.43%) in the loss condition, with no difference between conditions (*t*_(29)_ = −0.093, *p* = 0.926). For pupil diameter, one subject did not have sufficient quality data and was excluded from analysis. Pre-stimulus pupil diameter was taken as a measure of physiological effort (Figure 1D). Pupil diameter was significantly different between conditions (*F*_(2,56)_ = 16.55, *p* < 0.001), with larger diameter in both gain (*t*_(28)_ = −3.96, *p* < 0.001) and loss conditions (*t*_(28)_ = −4.92, *p* < 0.001), compared to the baseline condition, but no difference between the gain and loss conditions (*t*_(28)_ = −0.193, *p* = 0.849).

To analyze the development of performance and pupil diameter over the 10-min task duration (Time-on-Task), linear slope coefficients were calculated for each incentive condition. Both response speed and pupil diameter showed a gradual reduction over time-on-task (see Figures 1C,E). However this decline was not significantly different between conditions (performance: *F*_(2,59)_ = 2.206, *p* = 0.119; pupil: *F*_(2,56)_ = 1.198, *p* = 0.309).

#### Effort Discounting Task

Rewards were discounted with longer task duration (i.e., higher effort) in both the gain and loss conditions (see Figure 1G). The area under the discounting curve (AUC) was used as a summary metric for discounting (larger AUC denotes less discounting). Discounting AUC was smaller in the gain compared to the loss condition (*t*_(29)_ = −3.139, *p* = 0.0039), indicating that people discounted less in the loss condition (see Figure 1H).

#### Order Effects

As the different incentive conditions in both tasks were performed in separate runs (gain, loss, counter-balanced between-subjects), we tested for the potential effects of condition order. For PVT performance, a mixed ANOVA with Incentive (gain, loss) as within-subjects factor and Order (gain-loss, loss gain) as a between-subjects factor, yielded a significant Incentive × Order interaction (*F*_(1,59)_ = 9.54, *p* = 0.0045). Participants who performed the gain condition first, had better performance in the gain vs. the loss condition (*t*_(14)_ = 2.638, *p* = 0.0195), while participants who performed the loss condition first, performed slightly better (although non-significant) in the loss vs. the gain condition (*t*_(14)_ = −1.841, *p* = 0.087; see Supplementary Figure S2). Analysis of order effects in pupillometry did not yield a significant interaction (*F*_(1,57)_ = 0.189, *p* = 0.667).

Analysis of order effects in the Choice task yielded a significant Incentive × Order interaction (*F*_(1,59)_ = 6.20, *p* = 0.019), showing a strong loss aversion effect for participants who started with the gain condition (*t*_(14)_ = −3.367, *p* = 0.0046), but not for participants who performed the loss condition first (*t*_(14)_ = −0.953, *p* = 0.357; see Supplementary Figure S2).

### Discussion

Results of Experiment 1 indicated that gain and loss incentives were associated with better performance and larger pupil size on the sustained attention task, compared to the unincentivized condition. However, there was no difference in performance nor pupil diameter between the gain and the loss conditions during vigilance performance. In the discounting task, on the other hand, there was a significant difference between the gain and loss conditions. Participants were more willing to invest effort if they stood to lose money, compared to when an equivalent outcome was framed as a gain. These findings suggest that loss aversion influences effort investment during effort-based decision making, but not during the actual exertion of cognitive effort.

An important limitation, however, was that gain and loss conditions were presented in separate runs (both in vigilance and discounting tasks). Analysis of order effects indicated that loss aversion effects were different for participants who started with the gain condition vs. participants who started with the loss condition. To account for this, we conducted a second experiment in which loss and gains trials were intermixed on a trial-by-trial level.

## Experiment 2

### Methods

#### Participants and Procedure

We recruited an independent sample of 30 participants [mean age (stdev) = 21.90 (2.54), 16 females]. Like Experiment 1, participants performed a Motivated Vigilance Task, followed by a Discounting task. In contrast to Experiment 1, gain and loss trials were intermixed. After completion of the Discounting task, one choice trial was randomly drawn for execution.

#### Motivated Vigilance Task

**Figure 2:**
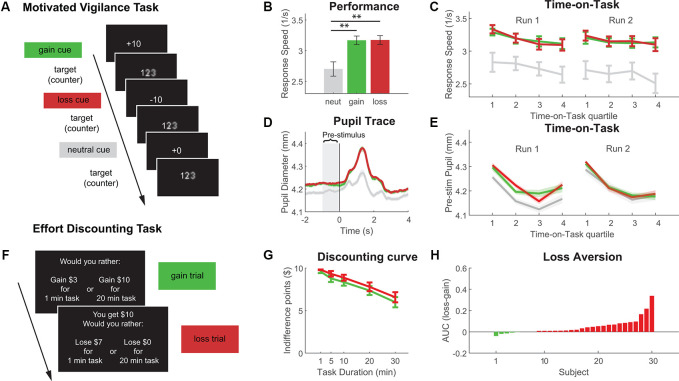
Results from Experiment 2 with **(A)** schematic of the Motivated Vigilance Task, **(B)** response speed in the vigilance task for baseline, gain and loss conditions, **(C)** Time-on-Task decline of response speed (Time-on-Task quartiles for illustrative purposes, statistical analysis was based on linear slopes), **(D)** target-locked pupil trace in the vigilance task (time = 0 indicates target presentation), **(E)** Time-on-Task decline of pre-stimulus pupil diameter (Time-on-Task quartiles for illustrative purposes only, statistical analysis was based on linear slopes), **(F)** example trials of the effort-discounting task, **(G)** effort-discounting curve, and **(H)** loss-gain difference in the area under the discounting curve (AUC; positive difference scores denote less discounting for loss vs. gain choices). **p < 0.01.

As in Experiment 1, the task started with an unincentivized baseline run, from which the median RT was extracted as a criterion for the subsequent runs. After this, participants performed two incentivized runs. Trials started with a reward cue indicating the incentive condition for that trial (Figure 2A). On gain trials, participants could win 10¢ if they responded faster than the criterion. On loss trials, they would lose 10¢ for responses slower than the criterion. On neutral trials, no incentives were given. Gain, loss, and neutral trials were pseudo-randomly intermixed, such that approximately equal numbers of trials from each condition were presented throughout the runs, and no more than three consecutive trials of the same incentive condition were presented. Participants completed two incentivized runs amounting to a total of approximately 60 trials per incentive condition.

#### Effort Discounting Task

The discounting task followed the same procedure as in Experiment 1, except that gain and loss trials were randomly intermixed (Figure 2F). While gain and loss trials were presented in intermixed fashion, adjusting staircase procedures updated the values for the gain and loss condition separately from trial to trial. Therefore, a separate set of indifference points was derived for gain and loss-framed decisions. The staircase procedure was repeated twice. Resulting indifference points were averaged for each effort level and incentive condition. As in Experiment 1, the discounting task was followed by the execution of one randomly drawn trial (see Supplementary Materials for full instructions).

### Results

#### Motivated Vigilance Task

Comparing response speed for gain, loss and neutral trials showed that there was a significant incentive effect (see Figure 2B; *F*_(2,58)_ = 14.23, *p* < 0.001), with responses faster in gain and loss trials compared to neutral trials (gain: *t*_(29)_ = −3.786, *p* < 0.001; loss: *t*_(29)_ = −3.781, *p* < 0.001), but no difference between gain and loss trials (*t*_(29)_ = −0.349, *p* = 0.729). In the gain condition 56.96% (±12.21%) responses were faster than criterion vs. 58.33% (±12.71%) in the loss condition (*t*_(29)_ = −0.739, *p* = 0.466). Eight participants did not have sufficient pupillometry data and were excluded from analysis. The remaining 22 subjects all had at least 23 trials with valid pupillometry data per incentive condition (see Figure 2D). Comparing pre-stimulus pupil diameter between incentive conditions showed that, there was a significant effect for incentive on pre-stimulus pupil diameter (*F*_(2,42)_ = 5.67, *p* = 0.007), with larger pupil size for gain and loss trials, compared to neutral trials (gain: *t*_(21)_ = −2.187, *p* = 0.044; loss: *t*_(21)_ = −2.928, *p* = 0.008), but no difference between gain and loss trials (*t*_(29)_ = −0.814, *p* = 0.425).

Time-on-Task effects were analyzed by calculating linear slope coefficients for each incentive condition in each run. As in Experiment 1, response speed and pupil diameter reduced over time-on-task (see Figures 2C,E), but there were no significant difference between incentive conditions (Run1 performance: *F*_(2,58)_ = 0.744, *p* = 0.480; Run1 pupil: *F*_(2,42)_ = 0.616, *p* = 0.545; Run2 performance: *F*_(2,58)_ = 0.038, *p* = 0.962; Run2 pupil: *F*_(2,42)_ = 1.21, *p* = 0.306).

#### Effort Discounting Task

Rewards were discounted with longer task durations (higher effort; see Figure 2G). Replicating Experiment 1, AUC was higher for loss compared to gain choices, indicating that participants discounted less strongly for losses than gains (*t*_(29)_ = −3.434, *p* = 0.0018; see Figure 2H).

### Discussion

Results from Experiment 2 fully replicated Experiment 1. During the performance of a vigilance task, both gain and loss incentives motivated higher effort exertion (performance and pupil diameter) compared to neutral, unincentivized trials. There was no difference between gain and loss trials, however. In contrast, participants did show loss aversion in the discounting task, as they were more willing to engage in further task performance if choices were framed as losses compared to gains. Importantly, as gain and loss trials were intermixed, these results could not be due to the influence of order effects. This demonstrates the robustness of the effects within the context of sustained attention. To further extend our findings to a different cognitive domain (working memory), we conducted a third experiment.

## Experiment 3

As cognitive tasks generally require mental effort, it is important to explore whether our findings about sustained attention extend to other domains. Working memory has been studied previously in the context of cognitive effort. A higher working memory load is experienced as more effortful (Bijleveld, 2018), and is associated with physiological and neural signs of increased effort (Kahneman and Beatty, 1966; Jansma et al., 2007; Richter et al., 2008), and effort-discounting (Westbrook et al., 2013, 2019). In this experiment, we examined whether effort allocation in the N-Back task would be differentially affected by gains and losses. Paralleling Experiments 1 and 2, we tested this both in the context of cognitive performance, and effort-based decision making. Since a larger number of N-Back levels needed to be sampled (1–4-Back), the N-Back performance and decision-making tasks were tested in two separate sub-experiments (Experiments 3a and 3b).

### Methods

#### Motivated N-Back Task (Experiment 3a)

**Figure 3:**
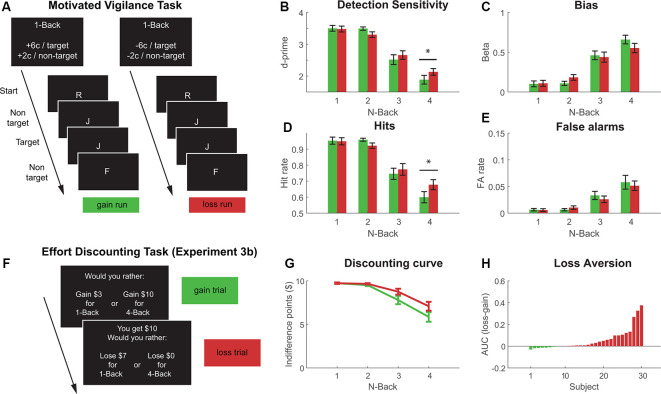
Results from Experiments 3a and 3b with **(A)** schematic of the incentivized N-Back task in Experiment3a, **(B)** detection sensitivity (d-prime) in the N-Back task for gain and loss conditions, **(C)** detection bias (beta), **(D)** hit rate, **(E)** false alarm rate, **(F)** example trials in the Effort Discounting Task in Experiment 3b **(G)** effort-discounting indifference points and **(H)** loss-gain difference in the area under the discounting curve (AUC; positive difference scores denote less discounting for loss vs. gain choices). *p < 0.05.

Thirty-two participants were recruited for this experiment [mean age (stdev) = 23.16 (3.27), 16 females]. Participants performed an N-Back task under four different levels of memory load (1, 2, 3, 4-Back; see Figure 3A). Participants were presented with a series of letter stimuli (1-s presentation, 3-s ISI), and had to respond with a target button press if the current letter matched the letter that was presented N positions before the current stimulus. If the current letter did not match the letter N positions back, a non-target button press was required. Each task run consisted of 64 stimuli, 16 of which were targets. The experiment started with a practice phase, in which all levels of N-Back were trained to criterion (>50% correct responses). Subsequently, participants performed two incentivized runs (gain and loss, order counter-balanced) for all N-Back levels. In the gain run, they could earn 2¢ for each correct non-target response and 6¢ for each correct target response. In the loss run, they received $2, and would lose 2¢ for each incorrect non-target response (or non-response), and lose 6¢ for each incorrect target response (or non-response). Given the target/non-target ratio, this incentive scheme neither biased towards more target nor non-target responses. Furthermore, a subjectively experienced effort was assessed after each incentivized run *via* a assessed after each incentivized run *via* a self-report scale (NASA-TLX).

#### Effort Discounting Task (Experiment 3b)

Thirty independent participants were recruited [mean age (stdev) = 22.53 (3.47), 16 females]. As in Experiment 3a, participants first completed a practice phase for all N-Back levels, after which the discounting task was performed (see Figure 3F). Participants were presented with a series of choice trials in which they were given the option between a lower variable amount of money in return for performing a low effort 1-Back, or a higher reward for performing a higher level N-Back (2, 3, 4-Back). In gain trials, higher rewards were fixed at $10, and lower rewards were variable between $0 and $10. In loss trials, participants were first instructed that they could receive $10 and that they would lose money for performing the low effort 1-Back, or lose $0 for performing the higher effort N-Back. Gain and loss trials were intermixed, and the monetary amount was dynamically updated following separate adjusting staircase procedures. Upon completion of all choice trials, one choice was randomly drawn for execution. Participants performed the chosen level of the N-Back task for a fixed duration of 15 min and received the associated reward. As in Experiments 1 and 2, the reward was not dependent on performance levels, but participants were instructed that they had to maintain effort throughout (see Supplementary Materials for full instructions).

### Results

#### Motivated N-Back Task (Experiment 3a)

A repeated-measures ANOVA with Effort Level (1, 2, 3, 4-Back), and Incentive Condition (Gain, Loss) yielded a significant main effect of Effort Level (*F*_(3,93)_ = 83.28, *p* < 0.001) on detection sensitivity (d-prime), but no main effect of Incentive Condition (*F*_(1,31)_ = 0.997, *p* = 0.326). Furthermore, there was a significant Effort Level × Incentive Condition interaction (*F*_(3,93)_ = 2.825, *p* = 0.043; see Figure 3B). Further deconstruction of this interaction showed no differences between gain and loss in detection sensitivity for the 1-Back (*t*_(31)_ = 0.192, *p* = 0.849), 2-Back (*t*_(31)_ = 1.93, *p* = 0.063), and 3-Back levels (*t*_(31)_ = −1.38, *p* = 0.178). However, for the 4-Back level, d-prime was significantly higher for the loss condition compared with the gain condition (*t*_(31)_ = −2.21, *p* = 0.034). Signal detection bias, on the other hand, increased with higher N-Back levels (*F*_(3,93)_ = 45.78, *p* < 0.001; see Figure 3C), with no difference between incentive conditions (*F*_(1,31)_ = 0.392, *p* = 0.536). Although bias was numerically higher in the Gain condition compared to the Loss for 4-Back condition, the Level × Incentive interaction did not reach significance (*F*_(3,93)_ = 2.371, *p* = 0.076). The increase in detection sensitivity at 4-Back level was primarily due to an increased hit rate for the loss condition (mean = 0.68, stdev = 0.18), compared to the gain condition (mean = 0.60, stdev = 0.20; *t*_(31)_ = −2.56, *p* = 0.016; see Figure 3D), but no difference in false alarm rate (gain: mean = 0.08, stdev = 0.097; loss: mean = 0.07, stdev = 0.067; *t*_(31)_ = 0.688, *p* = 0.497; see Figure 3E). Subjective effort did increase with higher N-Back levels (*F*_(3,87)_ = 22.81, *p* < 0.001), but not with incentive condition (main-effect: *F*_(1,29)_ = 1.29, *p* = 0.265; interaction: *F*_(3,87)_ = 0.41 *p* = 0.742).

#### Effort Discounting Task (Experiment 3b)

For decision making, reward value was discounted when higher levels of effort were required (higher N-Back levels; see Figure 3G. Furthermore, effort discounting was more pronounced for gain-framed decisions than loss-framed decisions (*t*_(29)_ = −3.091, *p* = 0.004; see Figure 3H), indicating robust loss aversion in effort-based decision-making.

### Discussion

Results from Experiment 3 replicated and extended findings in Experiments 1 and 2. In particular, participants were more willing to invest cognitive effort when decisions were framed as losses, rather than gains. Central to the aims of Experiment 3, the effort was operationalized as working memory load on an N-Back task. This indicates that the influence of loss framing on effort-based decision making generalizes across different cognitive domains (i.e., sustained attention and working memory). Results from Experiment 3a showed that loss incentives led to better cognitive performance at the highest effort level of the N-Back task (4-Back). At other N-Back levels, detection sensitivity was similar in the gain and loss conditions.

## Computational Modeling

**Figure 4:**
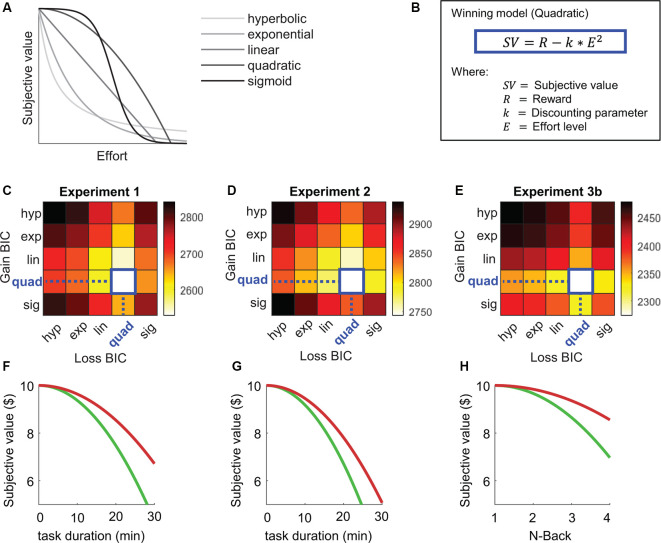
Computational modeling results with **(A)** illustration of different discounting models, **(B)** winning model (Quadratic), **(C–E)** model comparison using Bayesian Information Criterion (BIC) in Experiments 1, 2 and 3b, and **(F–H)** average discounting curves for Gain (green) and Loss (red) framing conditions in Experiments 1, 2 and 3b.

To characterize the shape of the discounting function underlying the choice data, five different discounting functions were fit to the individual choice data (see Figure 4A; hyperbolic, exponential, linear quadratic and sigmoid; Klein-Flügge et al., 2016; see Supplementary Materials for analysis details). Comparison of model fit indicated that in all three experiments the choice data were best modeled by a quadratic discounting function (see Figure 4B; Hartmann et al., 2013; Chong et al., 2016; Chen et al., 2020), both in the gain and in the loss conditions (see Figures 4C–H). Resulting discounting parameters (*k*) were compared between Gain and Loss conditions (square root transformed to correct for non-normality). Although average discounting rates were higher in the Gain compared to the Loss condition in all Experiments, this difference did not reach significance for Experiment 1 (*t*_(29)_ = 1.14, *p* = 0.265) and Experiment 2 (*t*_(29)_ = 1.77, *p* = 0.088). In Experiment 3b, however the Gain-Loss difference was significant (*t*_(29)_ = 2.357, *p* = 0.025). Moreover, when combining the samples across all experiments a significant Gain-Loss difference was confirmed (*t*_(89)_ = 2.645, *p* = 0.0097).

## General Discussion

We found that losses motivate cognitive effort expenditure more strongly than gains during decision-making. Loss aversion was consistently observed in all three effort-based decision-making experiments. Participants were more willing to invest effort when avoiding losses compared to when equivalent outcomes were framed as gains. This loss aversion effect may be dependent on the cognitive domain or the level of effort required.

### Effort-Based Decision Making Is Influenced by Loss Aversion

The first main finding of this study was that participants discounted loss-framed outcomes less than gain-framed outcomes. Individuals were willing to exert more effort to fend off a loss than to gain a reward. This loss aversion effect was present across cognitive domains (sustained attention and working memory), underlining the robustness of this effect. These results further expand the scope of loss aversion effects from risky and intertemporal decision making to effort-based choice. Only a few previous studies have explored the effects of loss aversion in effort-based decision-making. One study found that, in agreement with the current findings, people were more willing to invest the physical effort to avoid losses, compared to pursuing gains (Chen et al., 2020). Other studies, however, did not find such asymmetry (Nishiyama, 2016; Lockwood et al., 2017) or only in some populations (i.e., elderly, Byrne and Ghaiumy Anaraky, 2019). Importantly, the current effects were not confounded by delay or probability discounting, as these factors were strictly controlled. Moreover, computational modeling demonstrated that individual choice patterns were the best fit by a parabolic discounting function which has specifically associated with effort-discounting in previous studies (Hartmann et al., 2013; Chen et al., 2020).

### Losses Enhance Working Memory Performance Only at a High Cognitive Load

The effect of loss aversion effect on performance, however, was different for the sustained attention task vs. the working memory task. In Experiments 1 and 2, there was no difference in sustained attention performance for losses compared to gains. Concurrent pupillometry also showed no indications of higher effort in loss blocks (Experiment 1), or on loss trials (Experiment 2) compared to gains. In Experiment 3 on the other hand, losses were associated with better working memory performance only at the highest effort level (4-Back). These mixed findings concur with previous studies, some of which found no difference in performance between gain and loss conditions in a 3-Back task (Belayachi et al., 2015), while other studies found that loss incentives could even impair performance on other tasks (switch task, Stroop task, flanker task; Paschke et al., 2015; Carsten et al., 2019; Cubillo et al., 2019). Possibly, the effects of loss incentives may depend on the nature of the task (e.g., differentially affecting proactive vs. reactive control processes; Chiew and Braver, 2013; Botvinick and Braver, 2015).

Alternatively, the effects of loss aversion may only show at higher levels of effort. In Experiment 3a, working memory performance was not different between the gain and loss conditions on the lower effort levels (1–3-Back). Only at the highest effort level (4-Back) was performance enhanced for the loss compared to the gain condition. It is, therefore, possible that higher effort levels need to be probed before differential effects of gains and losses become apparent. Future studies could further explore the contributions of task type and effort level on the manifestation of loss aversion in cognitive performance.

### Conclusion

In total, this study shows that individuals are willing to invest more cognitive effort to avoid losses, compared to obtaining gains when making effort-based decisions. The effect of loss aversion effect on performance, however, may depend on the cognitive domain and/or task difficulty.

## Data Availability Statement

All data and code are available through the Open Science Framework: https://osf.io/fy9ms/.

## Ethics Statement

The studies involving human participants were reviewed and approved by National University of Singapore Institutional Review Board. The patients/participants provided their written informed consent to participate in this study.

## Author Contributions

SM designed the study, analyzed the data, and wrote the initial manuscript. ZP and CC programmed the experimental scripts, collected and analyzed the data, and edited the manuscript. MC was responsible for funding and revision of the manuscript.

## Conflict of Interest

The authors declare that the research was conducted in the absence of any commercial or financial relationships that could be construed as a potential conflict of interest.
