# Supplementary material for: Losses Motivate Cognitive Effort More Than Gains in Effort-Based Decision Making and Performance
Source: Front Hum Neurosci. 2020 Jul 17;14:287. doi: 10.3389/fnhum.2020.00287 (PMC7379863; doi:10.3389/fnhum.2020.00287)
Supplement: Supplementary file 1 [file Data_Sheet_1.pdf]

Supplementary materials

Supplementary materials for Massar, Pu, Chen, & Chee (2020). Losses motivate cognitive effort more than gains in effort-based decision making and performance. Front. Human. Neurosci.

Experiment 1

1. Tasks Instructions

Psychomotor Vigilance Task

The motivated Psychomotor Vigilance Task (PVT) is a sustained attention task that measures response speed over a 10-minute period (Dinges & Powell, 1985). Participants view a white fixation dot on a black screen. At random intervals (ISI 2-10 seconds; uniform distribution) the fixation dot is replaced by a running millisecond counter (target). Participants had to respond as quickly as possible to the appearance of the target. Upon response the counter will display RT for 1 second as performance feedback. Maintenance of performance in this task is found to be effortful, and to be correlated with reward motivation (Massar, Lim, Sasmita, & Chee, 2019; Massar, Lim, Sasmita, & Chee, 2016). In this study, participants completed three runs of the PVT. The first run was an unincentivized baseline run, used to determine the participant's individual response criterion for later (incentivized runs). The second and third runs were incentivized runs once under gain incentives, and once under loss incentives (order counterbalanced between subjects). Participants were given the below instructions.

Baseline run:

> "In this task you will be presented with a white dot at the center of the screen at the start of every trial. Your task is to fixate on that dot at all times and press the RIGHT CONTROL key with your RIGHT INDEX finger as soon as a stopwatch starts to run in place of the white dot. Upon pressing of the RIGHT CONTROL key, the stopwatch will stop and the number displayed on the screen will indicate your reaction time in milliseconds. You will perform 3 x 10 minutes of this PVT task. We will now start with a few trials. Press any key to proceed."

Gain run:

> "You will now begin another run of PVT for 10 minutes. For this run, you can earn some money based on your performance. For each trial with a reaction time less than XXXms (read off the screen), that means a faster reaction than XXXms, you will gain 10 cents; Since there will be roughly 80 trials, you can earn from $0 to about $8. Press any key to proceed."

Loss run:

> "You will now begin another run of PVT for 10 minutes. Before this run, you will be given $8 (Show participant $8 of cash and then put the cash somewhere near but visually inaccessible to the participant, e.g., another desk behind the participant, continue briefing). This $8 is yours but I am putting it here so that it does not distract you. However, during this

> run, you can lose some money from this $8 dependent on your performance. For each trial with a reaction time more than XXXms (read off the screen), that means a slower reaction than XXXms, you will lose 10 cents; Since there will be roughly 80 trials, you can lose from $0 to about $8 from this $8. Press any key to proceed."

Effort Discounting Task

Following the completion of the vigilance task, participants performed a choice task (effort discounting). In two runs, participants were presented choice trials in which they were offered a monetary rewards in return for further performance of the PVT for a given duration (gain condition), or the loss of an amount of money out of an initial endowment (loss condition; order counterbalanced between subjects). After all choices were made in both runs, one trial was randomly drawn for execution. Participants had to perform the PVT for the duration that they had chosen on that trial and were given the associated reward amount.

To ensure participants made their decisions based on their perceived effort of performing the task, and not on other decision factors (e.g. probability of receiving the indicated amount, temporal delay before receiving the amount), two instructions were provided. First, participants were instructed that the amount that they would receive, would not be dependent on their performance level (in contrast to the earlier incentivized PVT runs), but that they had to make their best effort to perform well. Second, to mitigate the influence of temporal delay to rewards on their choices, participants had to stay in lab for a fixed duration of 30 minutes after the choice task. During this time, they performed the PVT for the indicated duration and had a rest for the remaining time, after which they received their reimbursement. The maximum duration of 30-minutes was chosen, as participants would have completed three runs of the vigilance task (~30 minutes in total), prior to completing the discounting task. It could therefore be expected that participants had a reasonable grasp of how effortful performance of the task felt over time. Participants received the below instructions (for participants who performed the gain run first; instructions flipped when participant performed the loss run first).

Gain condition

> "In this round, which is the first round, you will encounter options like this (point at the example choice printed on the cover page of choice task questionnaire for the gain frame): would you a) do a 1-minute PVT and get $1 or b) do a 30-minute PVT and get $10. Your task is to choose the option you prefer to do after the choice task by pressing the left or right arrow key on the keyboard accordingly. Remember, if you were to do the PVT for less than 30 minutes, you are free to use the remaining of your time to do other activities. At the end of the choice task, the computer will randomly generate a pair of options you have previously seen and based on your previous choice, you will then need to perform the PVT task for the stated amount of time and the monetary outcome will be enforced 30 minutes after you start the PVT. It is therefore important for you to choose carefully during the choice task. Before we begin the choice task, please complete this questionnaire to test your understanding."
> *(Administer Choice Task Questionnaire for the gain condition, if corrected filled, leave the room to allow participant to start the task. If participants ask whether the monetary outcome is dependent on their performance in the last PVT, tell them "we need you to put in as much effort as you can but it is not dependent on your performance".)*

Loss condition

"This is the second round of the choice task. Everything is the same as the first round except you will encounter a different type of options, such as, during the last 30 minutes of the experiment (point at the example choice printed on the cover page of choice task questionnaire for the loss frame), if you are given $10, would you then a) do a 1-minute PVT and lose $5 out of the $10 or b) do a 20-minute PVT and lose nothing. Remember, the computer will randomly generate an option you have chosen across the two rounds of the task and the stated time of PVT and the monetary outcome will be enforced 30 minutes after you start the PVT."
(Administer Choice Task Questionnaire for the loss condition, if corrected filled, leave the room to allow participant to start the task. If participants ask whether the monetary outcome is dependent on their performance in the last PVT, tell them "we need you to put in as much effort as you can but it is not dependent on your performance".)

Participants' understanding of these instructions was checked prior to choice task commencement, via a short questionnaire. They were presented several outcome scenarios, and had to indicate the required task duration, rest duration, and reward amount. An example item is displayed below (the full questionnaire can be found on https://osf.io/fy9ms/).

Please indicate your answer by striking a line on the scale.

Scenario: 20 minutes of PVT and get $9

1. How long would you need to do the PVT for?

0 **30**

2. How long do you have to wait after the PVT?

0 **30**

3. How much is your monetary reward?

0 **10**

Figure S1. Example item from the instruction check questionnaire taken before starting the choice task.

2. Pupil analysis

During performance of the Psychomotor Vigilance Task, pupil diameter was continuously monitored (Tobii X60, Danderyd, Sweden). Pupil diameter was recorded from both eyes at a sampling frequency of 60Hz. Segments of missing data due to blinks or artefacts were interpolated. Resulting pupil traces were low-pass filtered (10Hz), and averaged between the right and left eye. Following earlier studies from our lab (Massar et al., 2019; Massar et al., 2016), we quantified the average diameter in a 1-second window preceding each target as an indication of tonic attentional effort (relevant for sustained attention. Trials with more than 50% interpolated data in the pre-stimulus window were excluded. This resulted in an average of 73.07 (± 11.06) trials in baseline, 75.14 (± 12.27) trials in Gain, and 70.38 (± 19.66) trials in loss. Comparison of number of valid trials between conditions did not show a significant differences (F(2,56) = 1.65, p = .2008).

3. Condition order effects

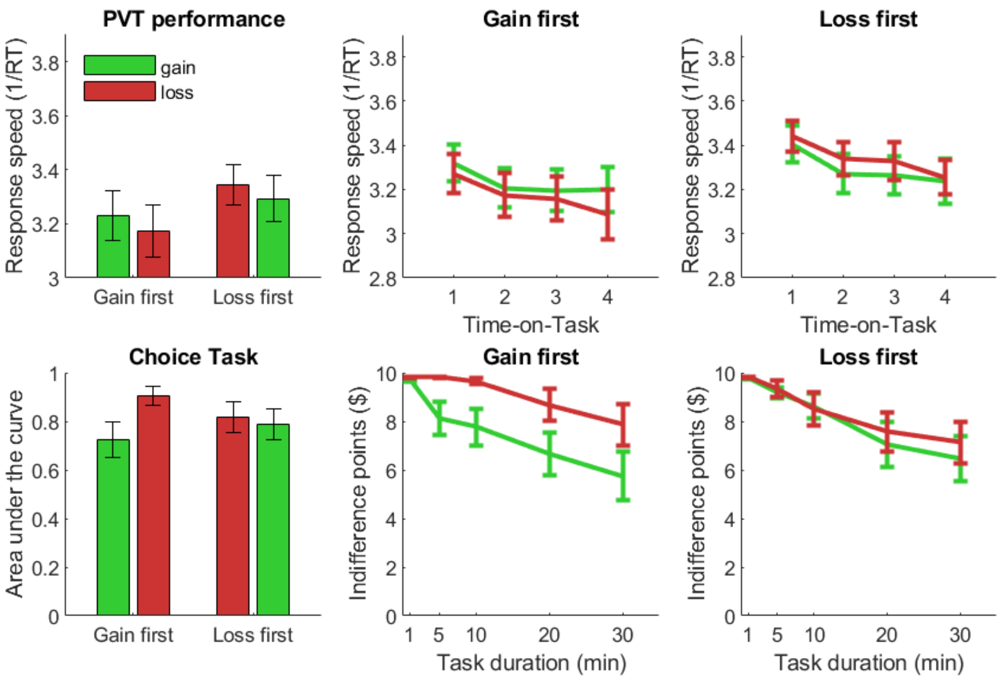

Figure S2. Data separated for participants who completed the Gain condition first versus those who completed the Loss condition first, for the Psychomotor Vigilance Task (PVT; upper panels), and the Choice Task (lower panels).

4. Correlation analysis

We next examined whether any differences between gain and loss conditions in performance and pupil diameter during the PVT task, were related with the individual's loss aversion as measured in the effort-based choice task. Individual loss aversion scores were calculated for performance (Response speed: loss-gain), pre-stimulus pupil diameter (loss-gain), and effort discounting (AUC: loss-gain), such that positive scores would indicate higher effort for loss compared to gain conditions. Unexpectedly, there was a significant negative correlation between performance loss aversion, and choice base loss aversion (r = -.401, p = .028). This correlation indicated that those participants who showed the largest improvement of performance in the loss PVT runs compared to the gain PVT runs, expressed the least increase in willingness to expand effort in the loss choice task compared to the gain choice task. While not in the expected direction, this correlation may be related to the condition order effects found. No significant correlation between pupil size and choice task loss aversion was found (r = .13, p = .49).

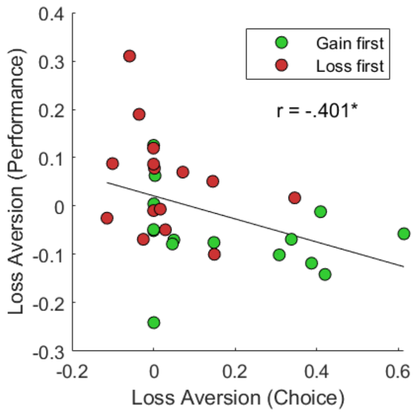

Figure S3. Correlations between loss aversion effects in the Choice task (AUC loss-gain), and PVT performance (response speed loss-gain).

Experiment 2

1. Tasks instructions

Psychomotor Vigilance Task

As in Experiment 1, participants first performed an unincentivized baseline run to establish the individual RT criterion (median RT in baseline). In this Experiment the baseline run lasted 5 minutes, after which two incentivized runs were performed. Critically, the incentivized runs included Gain trials, Loss trials and trials in which no incentive was offered (Neutral trials). All trial types were pseudo-randomly intermixed to ensure equal numbers of trials for each condition across the full duration of the incentivized runs. In total, incentivized runs lasted for approximately 12.75 minutes, including approximately 30 trials of each incentive condition. Between the first and second incentivized run, the individual RT criterion was updated to the median RT in the preceding run, to ensure comparable numbers of successful RTs below criterion (which were rewarded) across both runs. Participants received the below instructions.

Baseline run:

> "In this task you will be presented with a white dot at the center of the screen at the start of every trial. Your task is to fixate on that dot at all times and press the RIGHT CONTROL key with your RIGHT INDEX finger as soon as a stopwatch starts to run in place of the white dot. Upon pressing of the RIGHT CONTROL key, the stopwatch will stop and the number displayed on the screen will indicate your reaction time in milliseconds. You will perform 3 runs of this PVT task. We will now start with a few trials. Press any key to proceed.
>
> After I leave the room, you can proceed with the first PVT run, which will last for about 5 minutes. When you are done, please ring the call bell on the desk and I will come in and help you proceed."

Incentivized runs:

> "You will now begin a second/third PVT, which lasts for slightly less than 13 minutes. Before this run, you'll be given $5. The money is yours but I am putting it here so that it does not distract you. In the next run, before each trial, a symbol will appear at the center of the screen to offer you some incentive for a faster response in the upcoming trial. If you see +10c before a trial, it means if your target response in the upcoming trial is faster or equal to XXXms (read off screen), you will earn 10 cents. If not, there is no monetary consequence. If you see 0c, it means your response in the upcoming trial has no monetary consequence. If you see -10c, it means that if your response in the upcoming trial is slower than XXXms (read off screen), you will lose 10 cents. If not, there is no monetary consequence.
> Do you have any questions? … Let's start with some practice.
> *(Participants practice 6 trials. If they gain or lose money in any trial, provide feedback, e.g., "You just earned/lost 10 cents".)*
>
> After I leave the room, you can start the run. You do not have to start right now. Just start when you feel ready. Remember that the criterion is XXX ms."

Choice Task

Comparable to Experiment 1, the PVT was followed by an effort-based choice task, in which participants decided how to spend the last 30 minutes of the experiment. Choices again pitted short PVT performance for a lower reward against longer duration PVT performance for a higher reward. In contrast to Experiment 1, Gain and Loss choices were fully intermixed (presented in the same runs in alternating fashion). Participants received the below instructions (understand of which was checked using the same questionnaire as in Experiment 1).

> "We will now proceed to the choice task. At the end of the choice task, you will have 30 minutes left in the experiment. You will use this time to perform a PVT task, and if there is time left, other activities within your seat (you are free to use your phone, do your work, browse the internet, etc, but you cannot leave). The amount of time that you would need to do the PVT for will be determined based on your decisions in the choice task.
>
> In the choice task, you will be presented with a pair of options on either side of the screen for multiple trials. For example, … (show participants 4 example choices printed on the cover page of the choice task questionnaire, 2 in gain frame and 2 in loss frame.) Your task is to choose the option you prefer to do in the last 30 minutes of the experiment after the choice task. Remember, if you were to do the PVT for less than 30 minutes, you are free to use the remaining of your time to do other activities. At the end of the choice task, the computer will randomly generate a pair of options you have previously encountered and based on your choice between that pair, you will then need to perform the PVT task for the stated amount of time and the monetary outcome will be enforced 30 minutes after you start the PVT. It is therefore important for you to choose carefully during the choice task.
>
> For example, if you encounter this pair of options and choose the option on the left, and after the choice task this choice happens to be selected for implementation, then you will need to do 20 minutes of PVT and gain $9 30 minutes after you start the PVT. Alternatively, if you encounter this pair of options and choose the option on the right, and after the choice task this choice happens to be selected for implementation, then you will be given $7, do 5 minutes of PVT and lose nothing out of it 30 minutes after you start the PVT."
> *(Administer Choice Task Questionnaire and make sure participants answer correctly. If participants ask whether the monetary outcome is dependent on their performance in the last PVT, tell them "we need you to put in as much effort as you can but it is not dependent on your performance".)*

2. Pupil analysis

In Experiment 2, the same preprocessing procedures were followed as in Experiment 1. For reasons that are not entirely clear to us, pupil data for 8 participants was of low quality resulting in a loss of more than 60% of trials due to excessive artefacts/missing data. It is a known fact that eye-tracking quality can be low for some participants, potentially due to physical features such as occlusion of the pupil by drooping eyelids or dark eyelashes. In our experience this usually leads to exclusion of 6-7% of participants under normal conditions (Massar et al 2016 Exp 2&3; slightly higher under specific conditions such as sleep deprivation; Massar et al 2019). We are not entirely sure why the percentage was slightly higher in this experiment (N = 8/30 subjects, 27%), however, in order to ensure a sufficient number of trials for analysis, we excluded individuals who had more than 60% missing trials in any one condition (i.e. a minimum of 23 trials per condition). For the remaining N = 22 participants, the number of valid trials was 52.95 (± 9.28) for neutral trials, 54.64 (± 9.32) for Gain trials, and 54.00 (± 10.15) for Loss trials. No significant difference in the number of valid trials was found between incentive conditions ($F(2, 42) = 1.04$, $p = .361$).

3. Correlation

To examine the association between loss aversion in PVT performance/pupillometry and loss aversion in decision making, bivariate correlations were run. In contrast to Experiment 1, there were no significant correlations between choice loss aversion and performance (r = .118, p = .556), or pupil size (r = .182, p = .417).

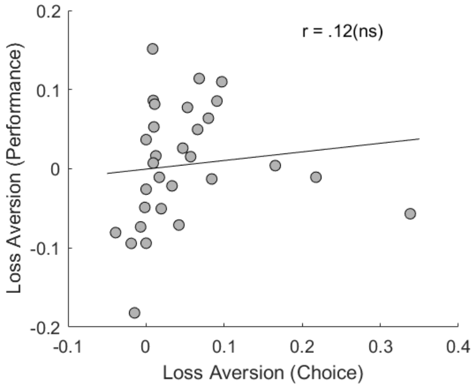

Figure S4. Correlations between loss aversion effects in the Choice task (AUC loss-gain), and PVT performance (response speed loss-gain).

Experiment 3a

1. Task instructions

Participants performed the N-Back task in four different effort levels (1-Back to 4-Back). They first practiced each level and had to reach 50% performance accuracy before proceeding to the next level. After having practiced all levels of the N-Back, participants performed an incentivized version of the N-Back in a Gain and in a Loss condition once for each N-Back level. N-Back levels were completed in ascending order (1-Back to 4-Back). The order of Gain and Loss runs were fully counterbalanced across all N-Back levels. This resulted in 16 unique Level x Incentive order sequences. Each participant was assigned to one of these orders, with a total of two participants per order (resulting sample size N=32). For the incentivized runs participants were given the below instructions.

Gain condition:

> "Here, you will perform the N-back task for money. In this block, every target for which you correctly press the "Y" key, you will receive 6 cents. Every non-target for which you correctly press the "N" key, you will receive 2 cents. Otherwise, you will receive zero cents. You can gain between $0 to $2 in this block. Do you have any questions?"
>
> *(If participants have questions, clarify the instructions so that they understand. Remind participants of the instructions for each N-back level, i.e. for 1-back, you are supposed to press "Y" if the current letter is the same as the previous letter; otherwise, press "N", etc. Instruct participants at every N-back level when you reach that level in the experimental task.)*

Loss condition:

> "Here, you will perform the N-back task for money. For this block, you will receive $2. Here is $2 [give $2 to participant.] Every target for which you press the wrong key (pressing "N" when you are supposed to press "Y") or if you don't press any key, you will lose 6 cents from the $2 that I am giving you. Every non-target for which you press the wrong key (pressing "Y" key when you are supposed to press "N") or if you don't press any key, you will lose 2 cents from the $2. Otherwise, you will lose zero cents. You can lose between $0 to $2 in this block. Do you have any questions?
>
> *(If participants have questions, clarify the instructions so that they understand. Remind participants of the instructions for each N-back level, i.e. for 1-back, you are supposed to press "Y" if the current letter is the same as the previous letter; otherwise, press "N", etc. Instruct participants at every N-back level when you reach that level in the experimental task.)*

Experiment 3b

1. Task instructions

An independent sample of participants was recruited in Experiment 3b. As in Experiment 3a, participants first practiced the N-Back task at all four levels (1-Back to 4-Back). Subsequently they completed an effort-based decision task in which they pitted performance of a low effort version of the task (1-Back) against a higher effort version (2-Back to 4-Back). At the end of the choice task, one trial was randomly drawn for implementation. The participant had to perform a 15-minute run of the N-Back task at the level that they had chosen on that trial, and they would receive the associated reward. Choice were either framed as Gain ("Would you rather do: a 1-Back for $2 or a 3-Back for $10") or as Loss ("You receive $10, would you rather do: a 1-Back and lose $8, or a 3-Back and lose $0"). Gain and Loss trials were pseudo-randomly intermixed across the task runs. Critically, participants were informed that their reward did not depend on the level of performance of the N-Back task, but that they were to uphold effort to perform, given a cover story that we could track their effort through their past performance and an eye-tracking device which was mounted on the desk (in actuality the eye-tracker was not activated). Participants received the below instructions.

> "We will now proceed to the choice task. Here, you will be presented with a pair of options on either side of the screen for multiple trials. For example, you may be shown this option…*(show printed examples and read examples out loud, i.e. "Would you rather do: a 3-back for $10 or a 1-back for $4?")*. Your task is to choose the option you prefer to do in the last 15 minutes of the experiment after the choice task. At the end of the choice task, the computer will randomly generate a pair of options that you have previously encountered and chosen. You will then perform the n-back task for the stated amount of time and monetary consequence. It is therefore important for you to choose carefully during the 'choice task' because each choice you make can potentially be implemented in the last 15 minutes of the experiment. And you will need to do the task for the entire 15 minutes. Do you have any questions?
>
> To choose the option on the left, press the left arrow and up arrow keys simultaneously. To choose the option on the right, press right arrow and up arrow keys simultaneously.
>
> Again, at the end of the choice task, you will perform the n-back task for an entire 15 minutes. During this final task, you will not be judged based on accuracy, but you will need to make every effort to do this task for the entire 15 minutes. We have ways of tracking your effort, based on your past levels of effort as well as an eye-tracking system that allows us to assess your attention levels. The number of n-back that you would need to do will be randomly selected from your decisions in this choice task, so choose carefully."

Computational modeling

To formally characterize the shape of the discounting functions underlying the choice data in the effort-based decision tasks, we used computational modeling. We fitted five different discounting functions that are known to be associated with discounting behaviour in different decision domains (e.g. temporal discounting, probability discounting, effort discounting). These functions were based on previous modelling literature from other labs (Klein-Flügge, Kennerley, Saraiva, Penny, & Bestmann, 2015) and ours (Massar et al., 2019).

Hyperbolic: Subjective values in temporal discounting follow a hyperbolic discounting function (Myerson & Green, 1995), which predicts that initial increases in duration result in larger decreases in subjective value. At longer durations, value reductions are thought to be smaller. The hyperbolic discounting function can be formalized as follows:

$$SV = \frac{R}{(1 + k * E)}$$

where *SV* is the subjective value, *R* is the reward magnitude, *E* is the effort level, and *k* is a free parameter denoting the individual discounting factor. Larger values of k would indicate steeper discounting.

Exponential: In contrast to hyperbolic discounting, the exponential discounting function assumes that the same increase in duration leads to the same percentage decrease in subjective value across time. This can be formalized as:

$$SV = R * e^{-k*E}$$

As in the hyperbolic model, *SV* is the subjective value, *R* is the reward magnitude, *E* is the effort level, and *k* is the individual discounting factor.

Linear: Simplest of all, the linear model describes a constant rate of reduction in SV independent of effort level. Larger *k* values indicate steeper rate of discounting. A key aspect of the linear model is that it can take negative subjective values at higher effort levels, where hyperbolic and exponential models are capped by an asymptote at SV = 0.

$$SV = R - k * E$$

Quadratic: Several studies examined the quadratic (or parabolic) model for effort-based decision making (Białaszek, Marcowski, & Ostaszewski, 2017; Chong et al., 2017; Hartmann, Hager, Tobler, & Kaiser, 2013). Like the linear model, the quadratic model can take negative values at higher effort levels. Additionally, it predicts that initial increases in effort level result in smaller decreases in *SV*. At higher effort levels (longer task durations, or high N-Bac levels), however, *SV* decreases with an increasingly steep slope, eventually converging to $-\infty$.

$$SV = R - k * E^2$$

Here, *k* describes the steepness of the discounting function.

Sigmoidal: We modelled our choice data using a sigmoidal function following Klein-Flugge et al. 2015. Like the quadratic model, the sigmoidal model assumes that initial increases in effort would only result in small reductions in subjective value, whereas at higher effort levels, there is a steep decline in subjective value. Unlike the quadratic model, however, the sigmoidal model does not assume negative values, as the subjective value approaches zero, limiting further value reductions.

$$SV = R * \left(1 - \left(\frac{1}{(1 + e^{-k*(E-p)})} - \frac{1}{(1 + e^{k*p})}\right) \cdot \left(1 + \frac{1}{e^{k*p}}\right)\right)$$

Here, *k* denotes the slope of the sigmoidal function, with smaller values of *k* indicating more gradual changes in *SV*. A second free parameter, *p*, indicates the inflection point (i.e. the effort level where *SV* has decreased to half the objective reward value *R*), and p is scaled to the levels of effort (here, minutes of PVT duration or N-Back level).

For all models, the probability of the observed set of choices was fitted using a softmax function. This can be formalized as:

$$P(choose\ Effort) = \frac{1}{(1 + e^{-\beta*(SV-Vss)})}$$

where *SV* is the subjective value of the effortful option as derived from the above models, and *Vss* is the objective reward value for the low effort option. The free parameter β is the temperature, indicating how tightly choice probability is based on the difference in value between both options. Smaller values of β, indicate more random choices.

Fitting procedure:

Summed Negative Log-Likelihood was calculated for each subject given the set of parameters and model to identify the combination of free parameters (*k*, *p*, and β in the sigmoidal model; *k*, and β in all other models) that produced the best fit to the data. For each subject and model, the set of parameters that produced the best fit were the ones that led to the least summed Negative Log-Likelihood, identified using MATLAB function fmincon. A lower bound of 0 was applied to parameter *k* in all models to ensure that as effort level increases subjective value decreases. To prevent parameter *k* from taking extreme values, an upper bound was applied depending on model and experiment (10 in hyperbolic and exponential models across 3 experiments, 2.5 in linear model and 0.625 in quadratic model in Experiments 1 and 2, 10 in linear and quadratic models in Experiment 3b, 1 in sigmoidal model in Experiments 1 and 2, 4 in sigmoidal model in Experiment 3b). A lower bound of 1 and an upper bound equal to twice the maximum effort level were applied to parameter *p* in the sigmoidal model (upper bound = 60 in Experiments 1 and 2, 8 in Experiment 3b). For all models and experiments the softmax β was varied between 0 and 1. To ensure that a global rather than local minimum of summed Negative Log-Likelihood was found for each subject given the model, fmincon was run 500 times, each time with a set of starting values (for parameters) randomly generated within their respective lower and upper bounds.

References

Białaszek, W., Marcowski, P., & Ostaszewski, P. (2017). Physical and cognitive effort discounting across different reward magnitudes: Tests of discounting models. *PLoS ONE, 12*(7), e0182353–0182326. doi:http://doi.org/10.1371/journal.pone.0182353

Chong, T. T.-J., Apps, M., Giehl, K., Sillence, A., Grima, L. L., & Husain, M. (2017). Neurocomputational mechanisms underlying subjective valuation of effort costs. *PLoS biology, 15*(2), e1002598-1002528. doi:10.1371/journal.pbio.1002598

Dinges, D. F., & Powell, J. W. (1985). Microcomputer analyses of performance on a portable, simple visual RT task during sustained operations. *Behavior Research Methods, Instruments, & Computers, 17*(6), 652–655. doi:http://doi.org/10.3758/BF03200977

Hartmann, M. N., Hager, O. M., Tobler, P. N., & Kaiser, S. (2013). Parabolic discounting of monetary rewards by physical effort. *Behavioural Processes, 100*, 192–196. doi:http://doi.org/10.1016/j.beproc.2013.09.014

Klein-Flügge, M. C., Kennerley, S. W., Saraiva, A. C., Penny, W. D., & Bestmann, S. (2015). Behavioral modeling of human choices reveals dissociable effects of physical effort and temporal delay on reward devaluation. *Plos Computational Biology, 11*(3), e1004116. doi:10.1371/journal.pcbi.1004116

Massar, S. A. A., Lim, J., Sasmita, K., & Chee, M. W. L. (2019). Sleep deprivation increases the costs of attentional effort: Performance, preference and pupil size. *Neuropsychologia, 123*, 169-177. doi:10.1016/j.neuropsychologia.2018.03.032

Massar, S. A. A., Lim, J., Sasmita, K. S., & Chee, M. (2016). Rewards Boost Sustained Attention through Higher Effort: A Value-based Decision Making Approach. *Biological Psychology, 120*, 21-27. doi:10.1016/j.biopsycho.2016.07.019

Myerson, J., & Green, L. (1995). Discounting of delayed rewards: Models of individual choice. *Journal of the Experimental Analysis of Behavior, 64*(3), 263–276. doi:10.1901/jeab.1995.64-263
